# Primary sarcoma of the pancreas, a rare histopathological entity. A case report with review of literature

**DOI:** 10.1186/1477-7819-9-85

**Published:** 2011-08-03

**Authors:** Peter Ambe, Christian Kautz, Shawqi Shadouh, Silvia Heggemann, Lothar K&#246hler

**Affiliations:** 1 Department of Surgery, St. Elisabeth Kreiskrankenhaus Grevenbroich, Akademisches Lehrkrankenhaus der RWTH Aachen, Germany; 2 Department of internal medicine and oncology, St. Elisabeth Kreiskrankenhaus Grevenbroich, Germany; 3 Institute of Pathology, St. Elisabeth Kreiskrankenhaus Grevenbroich, Germany; 4 Medical Faculty, RWTH - Aachen, Germany

## Abstract

**Aims:**

primary pancreatic sarcomas represent an extremely rare histopathological entity accounting for less than 0.1% of all pancreatic malignancies. Pancreatic sarcomas tend to be more aggressive and have a poor prognosis.

**Methods:**

the case of a 52 year old patient presenting with jaundice is presented and the available literature was reviewed.

**Results:**

primary pancreatic sarcomas are extremely rare. Pancreatic sarcomas are more aggressive than other pancreatic neoplasms.

**Conclusion:**

primary sarcomas of the pancreas are extremely rare, are aggressive and are associated with very poor prognosis.

## Background

Sarcomas represent a relatively rare malignant entity. Primary sarcomas of the pancreas are even rarer. Amongst pancreatic sarcomas, leiomyosarcomas have been most commonly reported. A review of the literature reveals seven cases of carcinosarcoma. We report a case of epitheloid carcinosarcoma in a young male patient presenting with pancreatitis and jaundice.

## Case presentation

**Figure 1:**
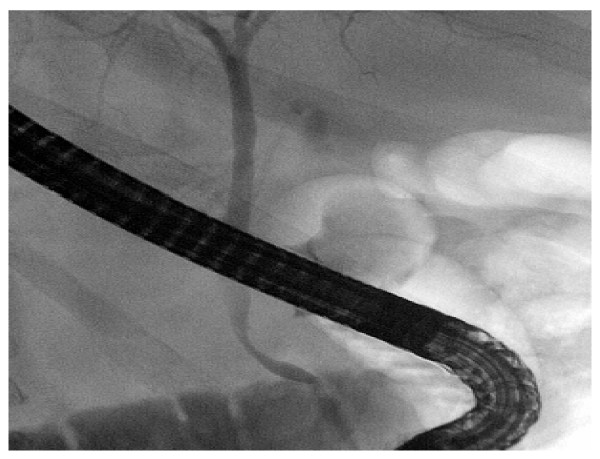
**ERCP at the initial presentation**. (normal common bile duct on ERCP at the initial presentation).

A 52 year old male with a history of chronic alcohol consumption was admitted in the medical department of our community hospital with an acute onset of upper abdominal pain, nausea and vomiting. The diagnostic workup revealed elevated amylase and lipase of 1012U/l and 1160U/l respectively. A swollen edematous pancreas caput and gallbladder stones were evident on upper abdominal ultrasound. At the time of admission, an endoscopic retrograde cholangio-pancreaticography (ERCP) was performed. The common bile duct however could not be visualized. ERCP was repeated after three days with papillotomy. A narrow common bile duct without stones or stenosis was visualized [Figure 1].

The patient recovered fully and was discharged after 8 days. One month after discharge the patient was readmitted with right upper quadrant pain and jaundice. The routine workup revealed elevated liver enzymes and bilirubin (total bilirubin: 10.78 mg/dl, direct bilirubin 9.86 mg/dl, indirect bilirubin:0.92 mg/dl, alkaline phosphatase: 337.64U/l). Cholecystitis was evident on upper abdominal sonography. Choledocholithiasis was present on ERCP, papillotomy and stone extraction were uneventful. Cholecystectomy was indicated.

**Figure 2:**
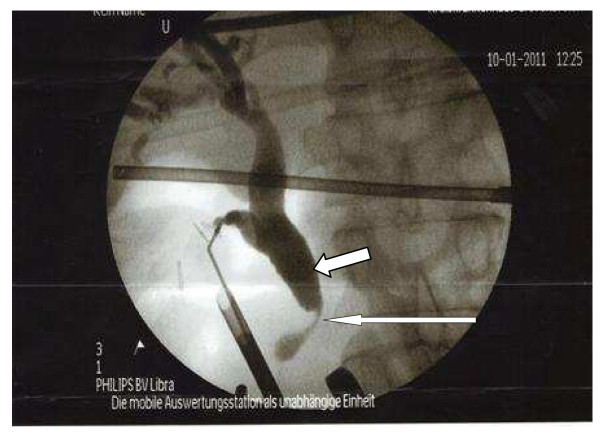
**Intraoperative Cholangiogramm**. (The thin arrow points at the stenosis, while the thick arrow demonstrates central dilated bile duct system).

**Figure 3:**
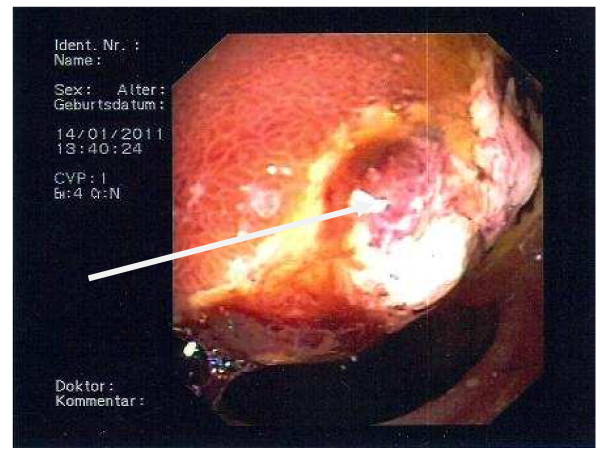
**Duodenal ulceration on ERCP**. (the arrow points at a 2 × 3 cm ulceration in the duodenum on ERCP).

**Figure 4:**
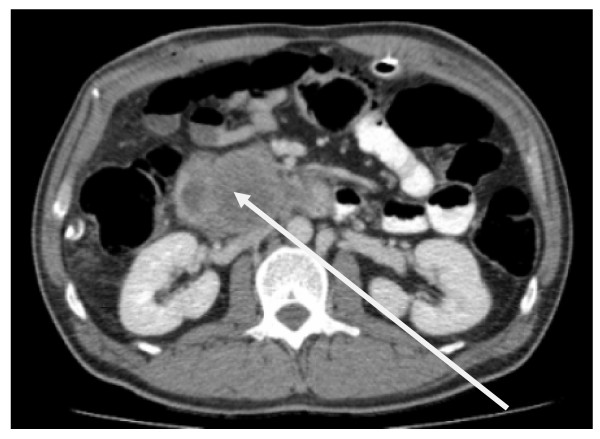
**Ct scan of the pancreas**. (The arrow marks the enlarged pancreatic caput).

Laparoscopic cholecystectomy was performed. During laparoscopy the main bile duct appeared unusally wide even after papillotomy and stone extraction, thus an intraoperative cholangiography was performed. This revealed a significant stenosis in the distal end of the choledochus. The proximal bile duct branches were dilated (Figure 2). An ERCP on day two after cholecystectomy showed a 2 × 3 cm measuring ulceration above the papilla of Vateri [Figure 3]. Biopsies revealed an ulcerating malignoma with duodenal infiltration. A stent was placed in the common bile duct. A CT scan of the abdomen revealed a large process of the pancreatic head without signs of mesenteric vessels infiltration [Figure 4].

On laparotomy a large tumor of the pancreatic caput enclosing the superior mesenteric and hepatic arteries with mesenterial infiltration was identified, making a complete resection unlikely. The tumor was left in situ and trans-duodenal biopsies were taken for histopathology. With a choledochus stent in place, a biliodigestive anastomosis was not indicated. To prevent future bowel obstruction, a gastroenteric anastomosis was constructed using small bowel 40 cm distal to the ligament of Treitz. The postoperative recovery was uneventful. A venous port system was implanted 10 days after laparotomy. The patient was discharged to our out-patient oncology. Chemotherapy with Gencitabine, 5-Fluorouracil and Folinic acid was initiated.

## Histopathology

**Figure 5:**
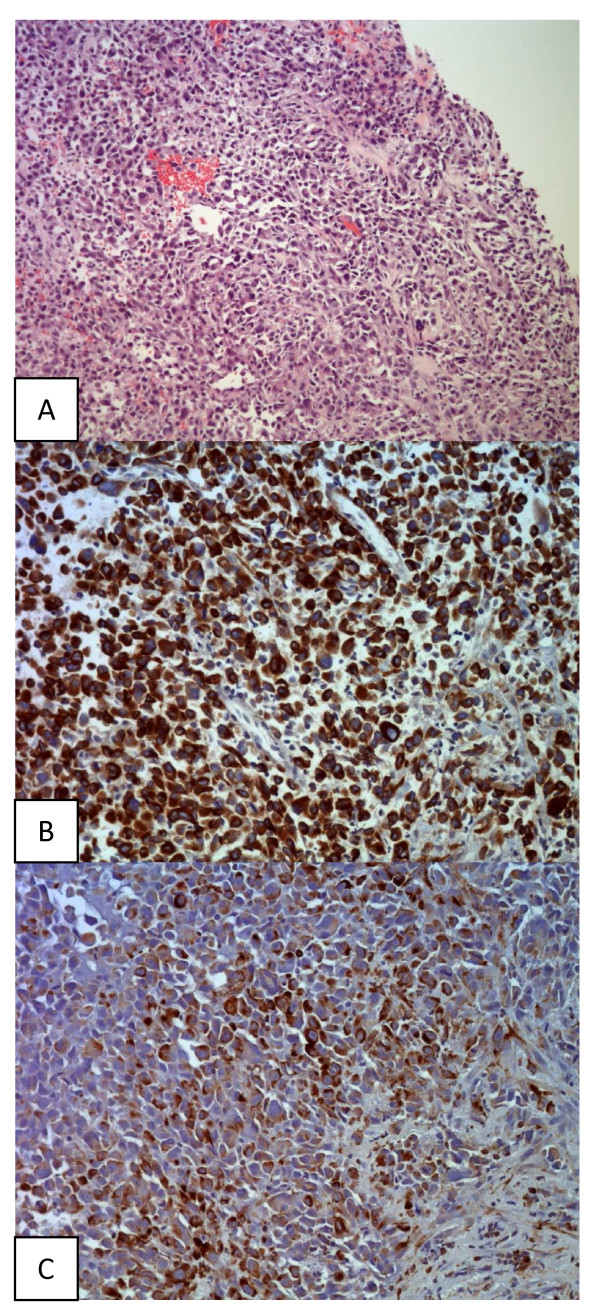
**Histological slides**. (HE, Cytokeratin and vemintin stains showing tumor cells with little or no pancreatic tissue).

Histologic sections revealed large neoplastic cells with epitheloid and sarcomatoid differentiation. Immunhistochemical staining demonstrated a co-expression of cytokeratin and vimentin. Stains for Caldesmon, CD 34, CD 31 and S100 were negative. A pure sarcoma or melanoma could be excluded on immunhistochemistry [Figure 5 A-C].

## Discussion

Sarcomas of the pancreas are exceedingly rare. Baylor et al reported a 0.1% incidence of pancreatic sarcoma after review 5000 cases of pancreatic cancer [1]. Amongst pancreatic sarcomas leiomyosarcomas tend to occur relatively frequent [2]. Epitheloid sarcomas also known as carcinosarcomas represent an even rarer subgroup with very few reported cases in the english literature. Darvishian and colleagues reported the 7^th ^case in the English literature [3]. Thus the incidence of carcinosarcoma as a single entity is expected to be far below 0.1%.

According to Feather et al sarcomas of the pancreas occur frequently in younger individuals. The pancreatic caput is most commonly involved followed by the tail and the body [4]. These features tend to correspond with those in the case presented. The patient, 52 years of age, relatively young, was diagnosed with a sarcoma of the pancreatic caput.

Clinically patients present with colicky pain, nausea and vomiting. These findings are basically similar to those of other pancreatic pathologies and are thus unspecific to sarcomas. Gray and many others consider a painless jaundice as a sign of an advanced lesion [5]. This was true for the case presented.

The diagnosis of pancreatic pathologies is usually confirmed using imaging techniques like ultrasound, computed tomography (Ct), magnetic resonance imaging (MRI), endoscopic retrograde cholangiopancreaticogrphy (ERCP) [6-8].

On initial presentation abdominal ultrasound and ERCP were not suspicious of a pancreatic malignancy as seen in figure 1. A pancreatic process was suspected on an intraoperative cholangiogramm as seen in figure 2. This was later confirmed on CT and ERCP. To what extend an early ct scan would have confirmed a malignancy remains unclear.

Surgical resection is the only possible cure for pancreatic malignancies. Radical resections are done for localized lesions. Advanced lesions warrant palliation. Potts et al proved the importance of a palliative gastric bypass in advanced stages [9].

In this case, the patient presented with an advanced tumor, so curative resection was not feasible. A palliative gastroenteric anastomosis was done. The common bile duct was not revised since a stent was placed during ERCP [10]. A venous port system was implanted and the patient was sent in for chemotherapy.

Pancreatic cancers generally have a poor prognosis since they tend to be diagnosed in an advanced stage. Sarcomas of the pancreas tend to grow much more rapidly and are believed to be associated with an even worse prognosis [4].

In the case presented, the sarcoma could have grown within three month, i.e between initial presentation in November 2010 and diagnosis in January 2011. This would support the notion that sarcomas tend to grow rapidly. The patient was discharged from the surgical department in a good shape and chemotherapy with Gencitabine, 5- FU and folinic acid was initiated.

## Conclusion

Primary sarcomas of the pancreas are extremely rare. Although little is known about pancreatic sarcomas, they appear to be more aggressive and are associated with a worse prognosis.

## Consent

Written informed consent was obtained from the patient for publication of this case report and accompanying images. A copy of the written consent is available for review by the Editor-in-Chief of this journal

## Conflict of interests statment

Drs. Ambe, Kautz, Shadouh, Köhler and cand med. Heggemann have no conflicts of interest or financial ties to disclose.

## Authors' contributions

PA, CK and SH did the literature research, PA wrote the article, SS did the pathology, LK edited the article. All the authors reviewed and approved the end version
